# Separation of ^44^Sc from Natural Calcium Carbonate Targets for Synthesis of ^44^Sc-DOTATATE

**DOI:** 10.3390/molecules23071787

**Published:** 2018-07-20

**Authors:** Krzysztof Kilian, Łukasz Cheda, Mateusz Sitarz, Katarzyna Szkliniarz, Jarosław Choiński, Anna Stolarz

**Affiliations:** 1 Heavy Ion Laboratory, University of Warsaw, Pasteura 5a, 02-093 Warsaw, Poland; msitarz@slcj.uw.edu.pl (M.S.); jch@slcj.uw.edu.pl (J.C.); anna@slcj.uw.edu.pl (A.S.); 2 Faculty of Chemistry, Biological and Chemical Research Centre, University of Warsaw, Żwirki i Wigury 101, 02-089 Warsaw, Poland; lcheda@chem.uw.edu.pl; 3 Faculty of Physics, University of Warsaw, Pasteura 5, 02-093 Warsaw, Poland; 4 Institute of Physics, Department of Nuclear Physics and its Applications, University of Silesia, ul. 75 Pułku Piechoty 1, 41-500 Chorzów, Poland; kasia.tworek@op.pl

**Keywords:** scandium-44, radiochemical separation, DOTATATE, solid target processing

## Abstract

The rapid increase in applications of scandium isotopes in nuclear medicine requires new efficient production routes for these radioisotopes. Recently, irradiations of calcium in cyclotrons by α, deuteron, and proton beams have been used. Therefore, effective post-irradiation separation and preconcentration of the radioactive scandium from the calcium matrix are important to obtain the pure final product in a relatively small volume. Nobias resin was used as a sorbent for effective separation of ^44^Sc from calcium targets. Separation was performed at pH 3 using a column containing 10 mg of resin. Scandium was eluted with 100 μL of 2 mol L^−1^ HCl. Particular attention was paid to the reduction of calcium concentration, presence of metallic impurities, robustness and simple automation. ^44^Sc was separated with 94.9 ± 2.8% yield, with results in the range of 91.7–99.0%. Purity of the eluate was confirmed with ICP-OES determination of metallic impurities and >99% chelation efficiency with DOTATATE, followed by >36 h radiochemical stability of the complex. A wide range of optimal conditions and robustness to target variability and suspended matter facilitates the proposed method in automatic systems for scandium isotope separation and synthesis of scandium-labeled radiopharmaceuticals.

## 1. Introduction

Due to their specific properties, interest in the positron-emitting scandium isotopes as supplementary PET isotopes has recently been observed. ^43^Sc (t_1/2_ = 3.89 h, branching ratio β^+^: 88%) and ^44^Sc (t_1/2_ = 3.97 h, branching ratio β^+^: 94.3%) are good alternatives to ^68^Ga, as they use similar complexing mechanisms. However, their half-lives are almost four times longer, which promotes applications for imaging processes, having slower pharmacokinetcs profiles. 

DOTA (1,4,7,10-tetraazacyclododecane-1,4,7,10-tetraacetic acid) is one of the most frequently used chelators acting as a conjugate between the radioisotope and a targeting molecule due to the quick and steady incorporation of the isotope and covalent bonds creation with biomolecules responsible for targeting. DOTA-octapeptides are a group of PET tracers that specifically bind to somatostatin receptors (SST) that are over-expressed on the neuroendocrine tumor (NET) cells. In particular, imaging of neuroendocrine tumors [1,2] exhibiting overexpression of somatostatin receptor type 2 (SSTR2) was promisingly demonstrated in some clinical and preclinical studies with somatostatine analogues [3,4,5].

The reason for the rapid increase in scandium applications is the development of new, efficient production routes for radioisotopes in cyclotrons by α, deuteron and proton irradiations. Methods where ^nat^CaCO_3_ was used as a target material have gained special attention due to the low cost of production. Effective production via the ^40^Ca(α,p)^43^Sc reaction was presented in Ref. [6], but the number of cyclotrons providing regular and intensive α beams is limited. Thus, proton irradiation with standard medical cyclotrons of ^44^Ca at its natural abundance (2.09%) in CaCO_3_ or CaO can provide adequate activity and be cost-effective for research and preclinical studies. Introduction of target material enriched with ^44^Ca allows to produce greater ^44^Sc activity for clinical studies and further regular applications, but due to the relatively high cost of ^44^CaCO_3_, the target material needs to be recovered [4]. For all cases, post-irradiation separation and preconcentration of the radioactive scandium from the calcium matrix is required to give the pure final product in a relatively small volume. Although calcium is non-toxic and is approved in radiopharmaceutical preparations, its excess could influence negatively the radiolabeling yield and, especially in the case of ^44^Ca, should be recovered for further use. Therefore, methods that allow effective scandium capture for labeling with the simultaneous release of possibly uncontaminated calcium for further processing are most often used [7]. For this purpose, filtration and solid phase extraction methods have been employed. In the first approach the target is dissolved in acid and neutralized to neutral or slightly alkaline conditions and scandium is separated as Sc(OH)_3_ precipitate on a 0.22 μm filter while calcium passes for further processing [8]. The chemical purity of the Sc product is important since the presence of other metals (Fe^3+^, Al^3+^, Zn^2+^) which form strong complexes with DOTA reduces the labeling yield, thus solid phase extraction on selective chelating or extracting sorbents was used. Ion exchange resin Chelex 100 [9], *N*,*N*,*N*′,*N*′-tetra-n-octyldiglicolamide (DGA) resin [4,10,11] or Uranium and Tetravalent Actinides (UTEVA) extraction resin [12] were used for minimizing metal impurities coming from processing the target or recovered material. Nobias PA-1 (iminobisacetic acid–ethylenediaminetriacetic acid chelate resin) was successfully applied in marine research, due to the fact that it has extremely low affinity for alkali metals and alkaline earth metals while on the other hand it shows high sorption of rare earth elements (REE) and was used for REE preconcentration and the elimination of alkali metals and alkaline earth metals in one step in seawater and salt water samples [13,14]. As trivalent scandium is comparable with trivalent REE with respect to chemical behavior, this work is focused on the experimental evaluation of Nobias as a sorbent for effective separation of ^44^Sc from calcium targets. Particular attention was paid to the reduction of the calcium matrix, the presence of metallic impurities, robustness, and simple automation. 

## 2. Results and Discussion

Natural calcium carbonate is an interesting target material for the production of diagnostic scandium isotopes for preclinical studies with Positron Emission Tomography (PET). Depending on the available infrastructure, reasonable activities of scandium-43 can be produced in the ^40^Ca(α,p)^43^Sc and ^40^Ca(α,n)^43^Ti→^43^Sc reactions, using heavy ion cyclotrons, or scandium-44 in the ^44^Ca(p,n)^44^Sc reaction by proton irradiation with medical cyclotrons of natural calcium containing 2.09% calcium-44. 

In the experiments, ^44^Sc was produced in reactions induced by 15 MeV protons. Twelve targets were irradiated resulting in 150–200 MBq at end of bombardment (EOB). 

Targets were placed in 10 mL polypropylene tubes, and 1 mL of 2 mol L^−1^ HCl was added. During dissolution, some differences in solubility were observed. Targets produced by protons of low beam intensity up to 10 μA dissolved quickly with intensive release of carbon dioxide. When working with targets irradiated with proton beam intensities higher than 10 μA (up to 25 μA), slower dissolution of the target material was observed due to the conversion of calcium carbonate to oxide. Complete dissolution of these targets required additional vortexing of the tube.

**Figure 1:**
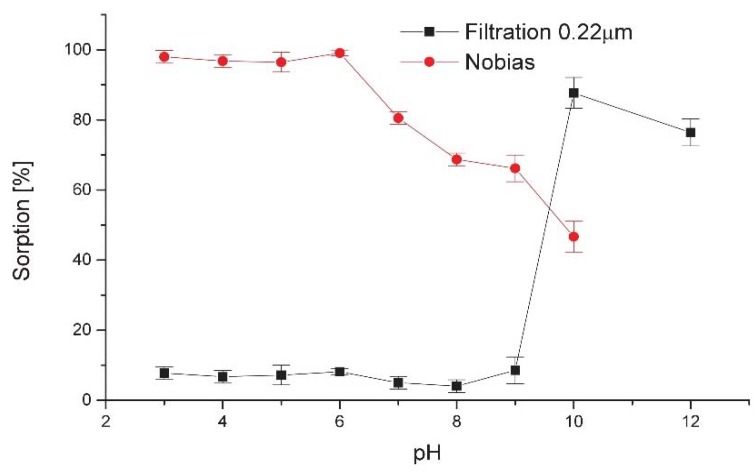
The effect of pH on the sorption of ^44^Sc(III) ions on 10 mg Nobias resin and precipitation on a 0.22 μm filter.

To evaluate the effect of pH on the sorption of scandium on Nobias resin, a series of sample solutions containing radioactive scandium ^44^Sc(III) at hundreds of kBq activities were adjusted to the pH range of 3.0–10.0 and processed according to the recommended procedure. During the separation process activity immobilized on column, activity of effluent and rinsing buffer, and eluted activity and residual activity on column after elution were measured with a wipe counter. The results obtained are given as sorption efficiency in Figure 1. For comparison, similar studies were performed for a 0.22 μm filter, which was recently applied to scandium separation by filtration [15]. The point at pH 12 was added to estimate the precipitate behavior at higher pH.

In the pH range of 3–6, sorption on the resin is almost quantitative, above 95%, and is stable and reproducible over the whole range. From pH 6 it decreases gradually but still exceeds 65%. Separation by precipitation on the 0.22 μm filter is effective at higher pH, reaching 90% at pH 10, but the optimum pH range is relatively narrow, sharply limited from pH 9 and gently decreasing at pH above 10. The results obtained are presented as sorption efficiency in Figure 1.

**Figure 2:**
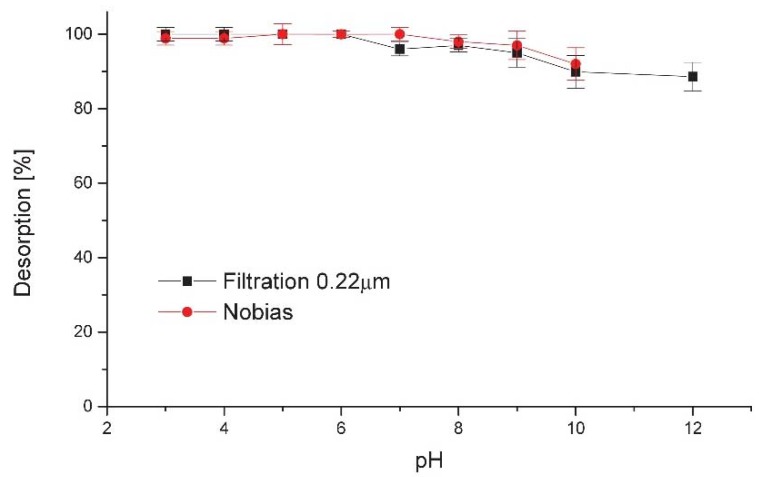
The effect of pH on the desorption of ^44^Sc(III) ions with 2 mol L^−1^ HCl on 10 mg Nobias resin and precipitation on a 0.22 μm filter.

To check the desorption efficiency and residual activity on the column or filter, the respective item was eluted with 0.5 mL of 2 mol L^−1^ hydrochloric acid and the ratio between initial column activity and eluate activity was calculated. The results obtained are presented as elution efficiency in Figure 2.

In the pH range of 3–6, the elution was complete and instantaneous, while there was a slight decrease in effectivity in more alkaline solutions, probably caused by slower precipitate dissolution kinetics than desorption in an acidic environment. This favors application of Nobias resin, where all operations are carried out under conditions that preclude the formation of precipitate. Slow desorption in alkaline media was also observed in [15], where stopped-flow elution from the filter was proposed for better recovery.

Both the narrow range of applicable pH and instability of elution conditions could explain the diversity in the efficiency of scandium separation procedures by filtration reported in the literature: 73% [8], 93.6% [16], and 96% Sc recovery [15]. These papers show some inconsistency in proposing optimal pH for precipitate formation from 6.5 to above 10 pH units. Our experience shows that the range is narrow and an optimal pH close to 10 is critical for effective separation. Experiments performed with a 0.22 μm Millex filter gave an efficiency of 73.3 ± 16.5%, with single results in the range 47.8–96.1%. The problems identified were pH fluctuations, time of eluent contact with the filter and clogging of the filter with precipitates and graphite particles from the target support, which significantly increased the back-pressure in the system and generated issues in automatic operation. Comparing with separation using Nobias resin, average separation efficiency was 94.9 ± 2.8%, with results in the range 91.7–99.0%. No backpressure effects were observed, and any graphite particles were immediately deposited on the top of the column without disturbing the flow. All the above results show that separation on Nobias could be more tolerant of any deviations in sample preparation or target variability.

Another important parameter is the final volume of eluate, as this affects the ability to obtain a solution with high specific activity. This parameter is critical for effective labeling, keeping control on the concentration of metallic impurities and for further preclinical application [10]. Scandium from the Nobias column was effectively and immediately eluted with 100 μL of 2 mol L^−1^ hydrochloric acid, giving a radioactivity concentration in the range 0.5–1.0 GBq/mL, depending on the initial activity, while for the 13 mm diameter Millex filter the minimum volume of eluent was 500 μL of 2 mol L^−1^ hydrochloric acid with 5 min stop-flow after rinsing the filter. In other filtration-based methods discussed above volumes of eluate start from 150 μL but representing only 55% of trapped activity [8], up to 3 mL of 6 mol L^−1^ HCl [16], where further removal of HCl excess was required. 

In a situation where recovery is necessary for economic reasons (i.e., for ^44^CaCO_3_ targets), the fraction collected after passing the sample through the Nobias column contains 98.1 ± 0.3% of the total calcium mass in the dissolved target, while a similar experiment for filtration gives 91.8 ± 0.6%. Subsequent rinsing with water increased this ratio to 99.8% and 99.7% respectively. 

Other separation methods used resins: UTEVA [12], DGA [4,10,11] or Chelex [9]. Comparison of the methods presented in Table 1 shows that separation using Nobias is comparable with the most effective methods proposed so far but consumes the smallest volume of eluent needed to wash the adsorbed isotope.

Chemical purity of the processed ^44^Sc solution is important, since the presence of other metals may interact with the DOTA chelator, as it is a non-specific complexing agent. Therefore, careful control of the metallic impurities in the sample is important for further labeling efficiency. The competing metals in the final solution were examined with a number of non-irradiated targets to estimate the concentrations of metal impurities using the ICP-OES method. The results presented in Table 1 show the concentrations of potential interferents at sub-mg L^−1^ levels, differing favorably from their content in other procedures where this effect was studied. This can be explained both by the use of high-purity reagents that are available for the proposed procedure and by the specific properties of the resin used. Nobias shows extremely low affinity towards alkaline metals [17] combined with almost quantitative sorption of transition metals in pH 5–6 [18] while the affinity is decreased significantly in pH < 4 [19,20]. REE metals show quantitative sorption in a similar pH range to the transition metals [21] but extend this property to more acidic conditions: pH 4.2 was successfully reported [13] and even pH 2.5 is suitable for efficient sorption [22], which is consistent for scandium in the present results (Figure 1). Thus, this pH value seems to be optimal, taking into consideration the sorption efficiency and simultaneous impurity removal.

As the reactivity of radioisotope and ligand is an indirect method of quantifying the metallic impurities and the final quality of the separated scandium, to check the overall method performance, radiolabeling with DOTATATE was used to asses and confirm the chemical purity of eluates. The radiochemical yields exceed 99% for 30 min reaction at 95 °C. The ^44^Sc-DOTATATE peak was observed at R_f_ = 0–0.2 in citrate buffer, while ^44^Sc^3+^ migrated with the front of the solution and a signal was recorded at R_f_ = 0.7–1.0. Reversed order was observed on the plates developed in ammonia acetate/methanol: unchelated Sc was deposited on the start line and ^44^Sc-DOTATATE traveled with R_f_ > 0.6. Maximum specific activity was 14 GBq/μmol but in routine experiments was set to 2.8 ± 0.3 GBq/μmol to standardize further steps. Both methods showed that labeling was effective and no deviations occurred due to poor quality of the Sc solution. Chromatograms and experimental results are available in Supplementary Materials.

Presence of metallic impurities in the labeling solution could cause the transmetallation and finally release of ^44^Sc^3+^ to the solution. Therefore, stability of labeled peptide could be thought as an indirect method of metallic contamination assessment. Proposed for DOTA-labeled peptides synthesis with ^44^Sc from generator [24] was adopted for cyclotron produced Sc [4,15] and referred to main impurity (Ca^2+^), metallic impurities behaving similarly to Sc^3+^ (Fe^3+^) or typical metallic impurities (Zn^2+^, Al^3+^, Ni^2+^, Cu^2+^), showing no significant changes in stability. To assess the transmetallation, a stability test, where ^44^Sc-DOTATATE was incubated for 36 h at room temperature with regular radiochemical purity determinations with HPLC was performed. To reduce the influence of the radiolytic decomposition of the peptide, mediated by γ-ray induced free radicals, 5% addition of ethanol as a scavenger was carried out. The results showed that transmetallation was negligible since the radiochemical purity did not drop below 99%, which confirmed the high stability of the ^44^Sc-DOTATATE labeling. For comparison another approach, based on determination of the lowest chelator concentration, required for quantitative labeling [25] was tested. Seven DOTATATE solutions, containing 0.07–28 nmol of peptide, were labeled with ^44^Sc under conditions previously successfully used, and the yield was then tested by TLC. All the samples gave satisfactory results, only for the lowest concentration yield decreased to 95.5%, showing the first signs of interferences in labeling, probably caused by cold metallic impurities competition. Comparable chelator amounts were used during DOTA labeling with ^44^Sc produced in cyclotron and separated on DGA resin (0.07 nmol), while labeling with ^44^Sc obtained from generator gave worse results and required at least 3 nmol [25] of chelator. Similar values were presented in another study for DOTATOC labeled with generator produced ^44^Sc, where more than 7 nmol of peptide was required for effective complexation [24], showing significant differences in labeling yields for ^44^Sc obtained from generator and cyclotron.

## 3. Materials and Methods

### 3.1. Chemicals

CaCO_3_ 99.999% trace metals basis (Merck, Darmstadt, Germany) was used for target preparation. Concentrated, high-purity hydrochloric acid (Traceselect^®^, Fluka, Honeywell Specialty Chemicals, Seelze, Germany) diluted to respective concentrations was used for dissolution of the target and elution of the column. Deionized water, purified using the MilliQsystem (SimplicityUV, Millipore, Burlington, MA, USA) was used for cleaning the labware and preparation of all solutions. The buffers were prepared from high purity formic acid (EMD Millipore, Merck Group, Darmstadt, Germany) in the pH range 2–3, acetic acid (TitriPUR^®^, EMD Millipore) in the range 4–5, sodium dihydrogen phosphate puriss.p.a. (EMD Millipore) from 6–7, sodium tetraborate puriss.p.a. (EMD Millipore) from 8–9 and ammonia solution from 10–12. Sodium hydroxide (TitriPUR^®^, EMD Millipore) was used for pH adjustment when required. 

Nobias Chelate-PA1 (Hitachi Technologies, Tokyo, Japan), methacrylate-based resin, functionalized with ethylenediaminetriacetic acid and iminodiacetic acid was used as a chelating resin. 

ICP multi-element standard solution for MS (10 mg L^−1^, EMD Millipore) was used for calibration standards in the determination of metallic impurities.

The ligand DOTATATE (DOTA-(Tyr3)-octreotate, C_65_H_90_N_14_O_19_S_2_ Mw= 1435.6 g/mol, Bachem, Bubendorf, Switzerland) was used as a model compound for labeling.

### 3.2. Apparatus

An ISMATEC peristaltic pump and 11 Elite (Harvard Apparatus, Holliston, MA, USA) syringe pump were used for liquid transfers, an Atomlab 500 dose calibrator and wipe tester (Biodex, Shirley, NY, USA) was used for activity determination, a Bioscan TLC reader Miniscan (Bioscan, Washington, DC, USA) with BioChrome software was used for thin layer chromatogram evaluations, a Thermo Scientific iCAP 6000ICP OES spectrometer was used for impurity determination. The instrumental settings of the manufacturers were as follows: RF generator power 1.15 kW, auxiliary gas flow 0.5 L min^−1^, nebulizer gas flow 0.40 L min^−1^, coolant gas flow 12 L min^−1^. For stability assessment the Shimadzu AD20 HPLC system with UV-Vis (ultraviolet-visible) and radiometric detector GabiStar (Raytest, Straubenhardt, Germany) was used.

### 3.3. ^44^Sc Production from ^nat^CaCO_3_

The target uses the design presented in [26]. Briefly, 85–90 mg of calcium carbonate was compacted in the form of a pill of ca. 6 mm diameter. The pill was then pressed into a graphite target support and placed in an alumina holder, fitting a home-made station for solid target irradiation (RP patent no. 227402) with a standard GE Pettrace 830 medical cyclotron. Targets were irradiated for around 2 h with 10 μA 15 MeV proton beam, resulting in 150–200 MBq activities at EOB.

### 3.4. ^44^Sc Separation

#### 3.4.1. Preconcentration System

**Figure 3:**
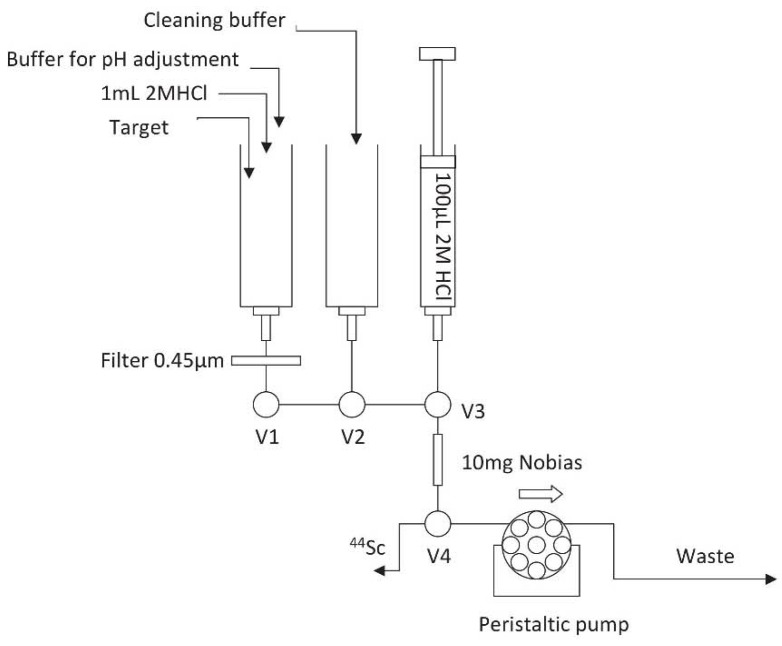
Scheme of the separation module. V1–V4—switching valves.

The preconcentration device (Figure 3) was equipped with a 3-valve module, common in many radiochemical devices i.e., Eckert-Ziegler. The target dissolution and reagent distribution were done in single sterile syringes screwed into the luer-lock connectors. Between the syringe and the column, a 0.45 μm filter was placed to remove any graphite particles, which could occur during target dissolution. The column was made in a 300 μL polypropylene pipette tip containing 10 mg of the resin placed between polyethylene frits. The sample and rinsing buffer were loaded with an ISM833 Ismatec peristaltic pump at 1 mL min^−1^, while the column was eluted at 0.25 mL min^−1^ with a micro-syringe pump to reduce the eluate volume and manifold blank. 

#### 3.4.2. Sorption Experiments

Targets were dissolved in 1 mL of hydrochloric acid and were aliquoted into volumes containing about 5% of target activity. To assess the sorption on Nobias, each portion was adjusted to a respective pH value from 3 to 10 with suitable buffers and sodium hydroxide, and then the solution was passed through the microcolumn. The column was rinsed with 3 mL of the buffer and activities of the column and eluate were measured with a dose calibrator. Elution of scandium was carried out using 0.5 mL of 2 mol L^−1^ HCl at a flow rate of 0.5 mL min^−1^, and the activity of the eluate and residual activity on the column were measured. 

#### 3.4.3. Separation by Filtration

To check the efficiency of scandium separation by precipitation and filtration, the sample was pH adjusted to a respective value from 3 to 12 pH units with suitable buffers and sodium hydroxide and the solution was loaded into a 0.2 μm filter (Whatmann, Buckinghamshire, UK) to trap any possible precipitate. Subsequently, the filter was rinsed with 3 mL of the buffer and the activities on the filter and in the eluate were measured with a dose calibrator. The scandium from the filter was eluted by 0.5 mL of 2 mol L^−1^ HCl at a flow rate of 0.5 mL minL^−1^ with a 30 s stop-flow after wetting the filter. The activity of the eluate and the residual filter activity were measured. Each experiment was done in triplicate. 

#### 3.4.4. Target Processing Procedure

**Figure 4:**
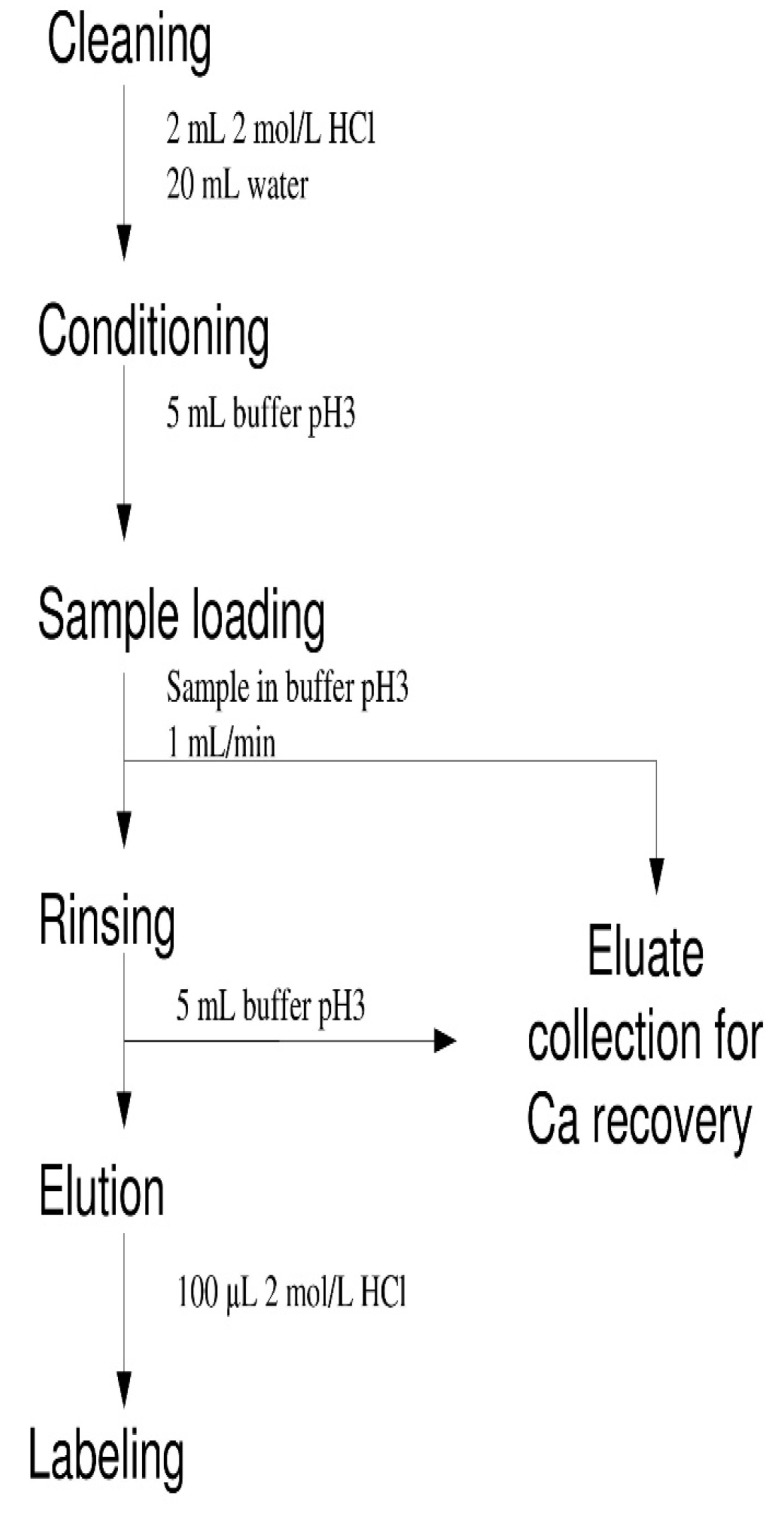
Flowchart of operations for target processing and scandium separation.

The irradiated target was disassembled with plastic tools and transferred to the syringe. 1 mL of 2 mol L^−1^ HCl was added and after complete dissolution of the calcium carbonate pellet 700 μL of 1 mol L^−1^ NaOH and 1 mL of 1 mol L^−1^ formic buffer pH 3.0 were added. Valve V1 was opened and the solution was pumped with a peristaltic pump through the column and the eluate was sent to wastes, where it could be collected separately for further recycling. Valve V1 was then closed and V2 was opened for rinsing the column with 2 mL of formic buffer pH 3.0. After evacuation of the solution V2 was closed, V4 was switched to another position, V3 was opened and the syringe pump was started, pumping 100 μL of 2 mol L^−1^ HCl, which was collected in the 1.5 mL Eppendorf tube for manual operations or injected directly to the reaction vial of the Eckert-Ziegler module for automated synthesis. The sequence of operations is presented on the flowchart in Figure 4. 

The entire process was performed with a number of non-irradiated targets to estimate the concentrations of metal impurities with ICP-OES. 

### 3.5. Chelation Efficiency

Radiolabeling of DOTATATE with scandium was performed by mixing the eluted scandium with 40 μg (ca. 28 nmol, 20 μL of 2 μg μL^−1^ stock solution in water) DOTATATE in 200 μL 0.2 mol L^−1^ ammonium acetate buffer (pH 4.0) and equimolar vs. added HCl amount of NaOH then heating for 30 min at 95 °C. Radiochemical yield was determined by thin layer chromatography by developing of 2–5 μL spots on 10 cm silica gel strips in 0.1 mol L^−1^ citrate buffer pH 4.0 or 1 mol L^−1^ ammonia acetate:methanol (1:1, *v/v*). Radioactivity distribution was recorded on a Bioscan TLC reader Miniscan and quantitatively evaluated with BioChrome software. For stability assessment the Shimadzu AD20 HPLC system with UV-Vis and GabiStar radiometric detector was used. The separation was done on a Phenomenex Gemini C18 column (150 mm × 4.0 mm i.d., 5 μm), with 375:115:0.5 (*v/v/v*) water:acetonitrile:trifluoroacetic acid as a mobile phase and 1.5 mL/min flow rate.

## 4. Conclusions

A fast and simple method of scandium-44 separation from natural calcium carbonate on Nobias chelating resin was presented. Almost quantitative separation, followed by high chemical purity and low final volume, favors this method for routine processing of solid targets. A wide range of optimal conditions and robustness to target variability and suspended matter facilitates the application of the method in automatic systems for scandium isotope separation and synthesis of scandium labeled radiopharmaceuticals.

## Figures and Tables

**Table 1:** Comparison of the methods used for separation of scandium radioisotopes from calcium carbonate targets.

**Resins**				
**Resin**	**Separation Efficiency (%)**	**Eluent/Final Volume (μL)**	**Impurities (mg L^−1^)**	**Ref**
DGA + Dowex 50	75	1 mol L^−1^ NH_4_OAc/1500	Pb < 0.7, Al < 1, Zn < 1, Cu < 0.02	[4]
DGA + SCX	93	NaCl/HCl/700		
UTEVA	80	H_2_O/400	N/A	[5]
Chelex 100	70	1 mol L^−1^ HCl/500	N/A	[9]
DGA	88 ± 3	0.1 mol L^−1^ HCl/10,000	Fe 1.14, Al 1.14	[10]
UTEVA	80 ± 4	H_2_O/1 mol L^−1^ HCl/400	Ca 82, Fe 5.2, Zn 4.7, Ni 29, Al. 6.4, Mn 2.0	[12]
DGA + SCX	89.7 ± 3.1	4.8 mol L^−1^ NaCl + 0.13 mol L^−1^ HCl/700	N/A	[23]
Nobias	94.9 ± 2.8	2 mol L^−1^ HCl/100	Al 0.009, Ca 0.34, Cu 0.02, Fe 0.005, Mn 0.014, Ni 0.013, Pb 0.03, Zn 0.03	This work
**Filtration**				
**Filter**	**Separation Efficiency (%)**	**Eluent/Final Volume (μL)**	**Impurities (mg/L)**	**Ref**
0.22 μm filter	96	0.5 mol L^−1^ HCl/N/A	Ca < 1, Fe 0.07	[15]
0.22 μm filter	93.6 ± 3.9	6 mol L^−1^ HCl/3000	N/A	[16]

## Supplementary Materials

The following are available online. Figure S1: pH dependence of Ca concentration in ^44^Sc solution after separation, Figure S2: Elution profiles of Nobias column and 0.22 μm filter, Figure S3: Thin-layer chromatograms of ^44^Sc-DOTATATE, ^44^Sc and resolution test for the mixture of ^44^Sc and ^44^Sc-DOTATATE, Figure S4: HPLC Resolution test for ^44^Sc and ^44^Sc-DOTATATE, Figure S5: Stability test for ^44^Sc-DOTATATE within 36 h after end of synthesis (EOS), Figure S6: γ-spectra of produced ^44^Sc, Table S1: ^44^Sc labeling yields for DOTATATE at different ligand concentrations.

## References

[1] Barrio M., Czernin J., Fanti S., Ambrosini V., Binse I., Du L., Eiber M., Herrmann K., Fendler W.P. (2017). The impact of somatostatin receptor-directed PET/CT on the management of patients with neuroendocrine tumor: A systematic review and meta-analysis. J. Nucl. Med..

[2] Heidari P., Kunawudhi A., Martinez-Quintanilla J., Szretter A., Shah K., Mahmood U. (2018). Somatostatin receptor type 2 as a radiotheranostic PET reporter gene for oncologic interventions. Theranostics.

[3] Singh A., Van Der Meulen N.P., Müller C., Klette I., Kulkarni H.R., Türler A., Schibli R., Baum R.P. (2017). First-in-Human PET/CT Imaging of Metastatic Neuroendocrine Neoplasms with Cyclotron-Produced 44Sc-DOTATOC: A Proof-of-Concept Study. Cancer Biother. Radiopharm..

[4] van der Meulen N.P., Bunka M., Domnanich K.A., Müller C., Haller S., Vermeulen C., Türler A., Schibli R. (2015). Cyclotron production of 44Sc: From bench to bedside. Nucl. Med. Biol..

[5] Hernandez R., Valdovinos H.F., Yang Y., Chakravarty R., Hong H., Barnhart T.E., Cai W. (2014). 44Sc: An attractive isotope for peptide-based PET imaging. Mol. Pharm..

[6] Walczak R., Krajewski S., Szkliniarz K., Sitarz M., Abbas K., Choiński J., Jakubowski A., Jastrzębski J., Majkowska A., Simonelli F. (2015). Cyclotron production of 43Sc for PET imaging. EJNMMI Phys..

[7] Pęgier M., Kilian K., Pyrzyńska K. (2018). Enrichment of scandium by carbon nanotubes in the presence of calcium matrix. Microchem. J..

[8] Severin G.W., Engle J.W., Valdovinos H.F., Barnhart T.E., Nickles R.J. (2012). Cyclotron produced 44gSc from natural calcium. Appl. Radiat. Isot..

[9] Krajewski S., Cydzik I., Abbas K., Bulgheroni A., Simonelli F., Holzwarth U., Bilewicz A. (2013). Cyclotron production of 44Sc for clinical application. Radiochim. Acta.

[10] Alliot C., Kerdjoudj R., Michel N., Haddad F., Huclier-Markai S. (2015). Cyclotron production of high purity 44m,44Sc with deuterons from 44CaCO_3_ targets. Nucl. Med. Biol..

[11] Müller C., Bunka M., Reber J., Fischer C., Zhernosekov K., Türler A., Schibli R. (2013). Promises of cyclotron-produced 44Sc as a diagnostic match for trivalent β-emitters: In vitro and in vivo study of A 44sc-dota-folate conjugate. J. Nucl. Med..

[12] Valdovinos H.F., Hernandez R., Barnhart T.E., Graves S., Cai W., Nickles R.J. (2015). Separation of cyclotron-produced 44Sc from a natural calcium target using a dipentyl pentylphosphonate functionalize dextraction resin. Appl. Radiat. Isot..

[13] Hatje V., Bruland K.W., Flegal A.R. (2014). Determination of rare earth elements after pre-concentration using NOBIAS-chelate PA-1®resin: Method development and application in the San Francisco Bay plume. Mar. Chem..

[14] Watanabe T., Saito-Kokubu Y., Murakami H., Iwatsuki T. (2018). Onsite chelate resin solid-phase extraction of rare earth elements in natural water samples: Its implication for studying past redox changes by inorganic geochemistry. Limnology.

[15] Misiak R., Walczak R., Wąs B., Bartyzel M., Mietelski J.W., Bilewicz A. (2017). 47Sc production development by cyclotron irradiation of 48Ca. J. Radioanal. Nucl. Chem..

[16] Minegishi K., Nagatsu K., Fukada M., Suzuki H., Ohya T., Zhang M.-R. (2016). Production of scandium-43 and -47 from a powdery calcium oxide target via the nat/44Ca(α,x)-channel. Appl. Radiat. Isot..

[17] Wang B.-S., Lee C.-P., Ho T.-Y. (2014). Trace metal determination in natural waters by automated solid phase extraction system and ICP-MS: The influence of low level Mg and Ca. Talanta.

[18] Minami T., Konagaya W., Zheng L., Takano S., Sasaki M., Murata R., Nakaguchi Y., Sohrin Y. (2015). An off-line automated preconcentration system with ethylenediaminetriacetate chelating resin for the determination of trace metals in seawater by high-resolution inductively coupled plasma mass spectrometry. Anal. Chim. Acta.

[19] Giakisikli G., Ayala Quezada A., Tanaka J., Anthemidis A.N., Murakami H., Teshima N., Sakai T. (2015). Automatic on-line solid-phase extraction-electrothermal atomic absorption spectrometry exploiting sequential injection analysis for trace vanadium, cadmium and lead determination in human urine samples. Anal. Sci..

[20] Quéroué F., Townsend A., Van Der Merwe P., Lannuzel D., Sarthou G., Bucciarelli E., Bowie A. (2014). Advances in the offline trace metal extraction of Mn, Co, Ni, Cu, Cd, and Pb from open ocean seawater samples with determination by sector field ICP-MS analysis. Anal. Methods.

[21] Zhu Z., Zheng A. (2018). Fast determination of yttrium and rare earth elements in seawater by inductively coupled plasma-mass spectrometry after online flow injection pretreatment. Molecules.

[22] Takata H., Tagami K., Aono T., Uchida S. (2009). Determination of trace levels of yttrium and rare earth elements in estuarine and coastal waters by inductively coupled plasma mass spectrometry following preconcentration with NOBIAS-CHELATE resin. Atom. Spectrosc..

[23] Domnanich K.A., Eichler R., Müller C., Jordi S., Yakusheva V., Braccini S., Behe M., Schibli R., Türler A., van der Meulen N.P. (2017). Production and separation of 43Sc for radiopharmaceutical purposes. EJNMMI Radiopharm. Chem..

[24] Pruszyński M., Majkowska-Pilip A., Loktionova N.S., Eppard E., Roesch F. (2012). Radiolabeling of DOTATOC with the long-lived positron emitter ^44^Sc. Appl. Radiat. Isot..

[25] Kerdjoudj R., Pniok M., Alliot C., Kubíček V., Havlíčková J., Rösch F., Hermann P., Huclier-Markai S. (2016). Scandium(III) complexes of monophosphorus acid DOTA analogues: A thermodynamic and radiolabelling study with 44Sc from cyclotron and from a ^44^Ti/^44^Sc generator. Dalton Trans..

[26] Stolarz A., Kowalska J.A., Jastrzębski J., Choiński J., Sitarz M., Szkliniarz K., Trzcińska A., Zipper W. (2018). Targets for production of the medical radioisotopes with alpha and proton or deuteron beams. AIP Conf. Proc..

