# Supplementary material for: Separation of 44Sc from Natural Calcium Carbonate Targets for Synthesis of 44Sc-DOTATATE
Source: Molecules. 2018 Jul 20;23(7):1787. doi: 10.3390/molecules23071787 (PMC6100303; doi:10.3390/molecules23071787)
Supplement: Supplementary file 1 [file molecules-23-01787-s001.pdf]

Supplementary materials

Separation of ^{44}Sc from natural calcium carbonate targets for synthesis of ^{44}Sc-DOTATATE

Krzysztof Kilian, Łukasz Cheda, Mateusz Sitarz, Katarzyna Szkliniarz, Jarosław Choiński, Anna Stolarz

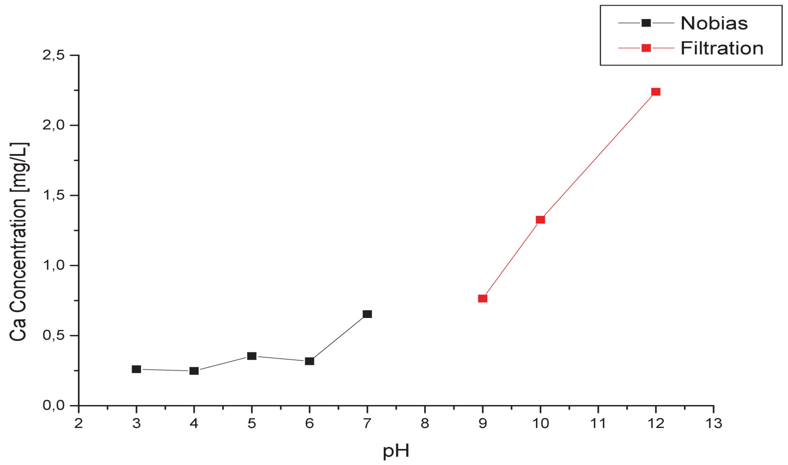

Figure 1S. pH dependence of Ca concentration in ^{44}Sc solution after separation

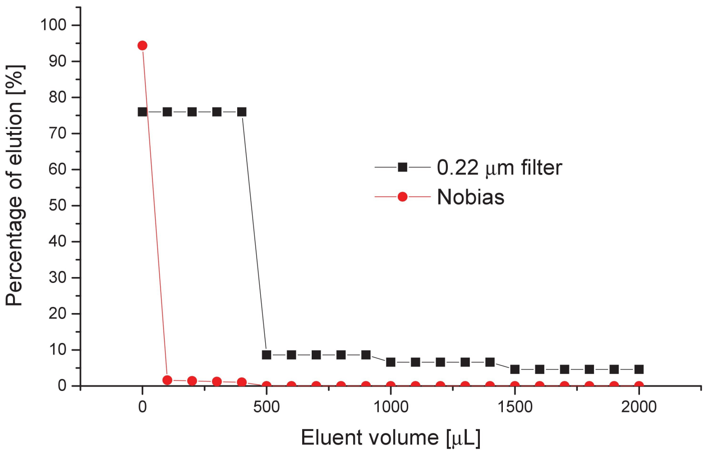

Figure 2S. Elution profiles of Nobias column and 0.22 μm filter

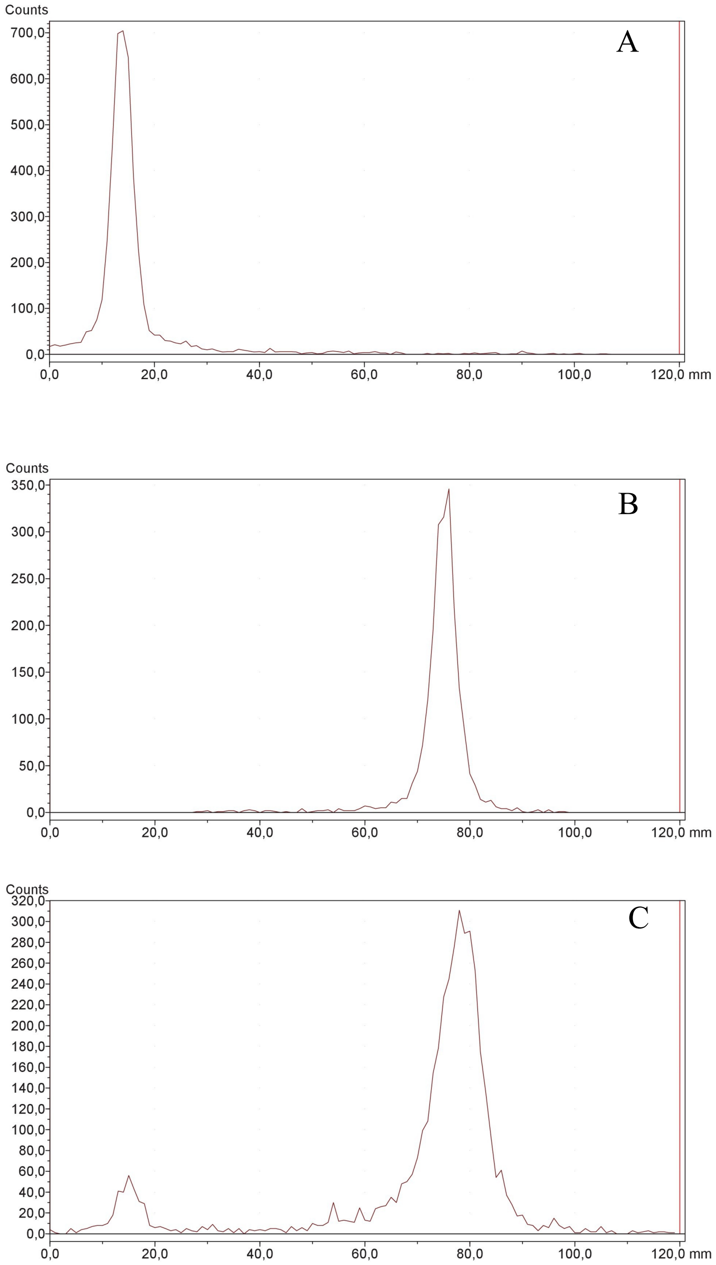

Figure 3S. Thin-layer chromatograms of **a)** ^{44}Sc-DOTATATE, R_f=0.0-0.2**; b)** ^{44}Sc R_f=0.7-1.0; c) resolution test for the mixture of ^{44}Sc and ^{44}Sc-DOTATATE. Citrate buffer 0.1 mol L^{-1}, pH=4.0.

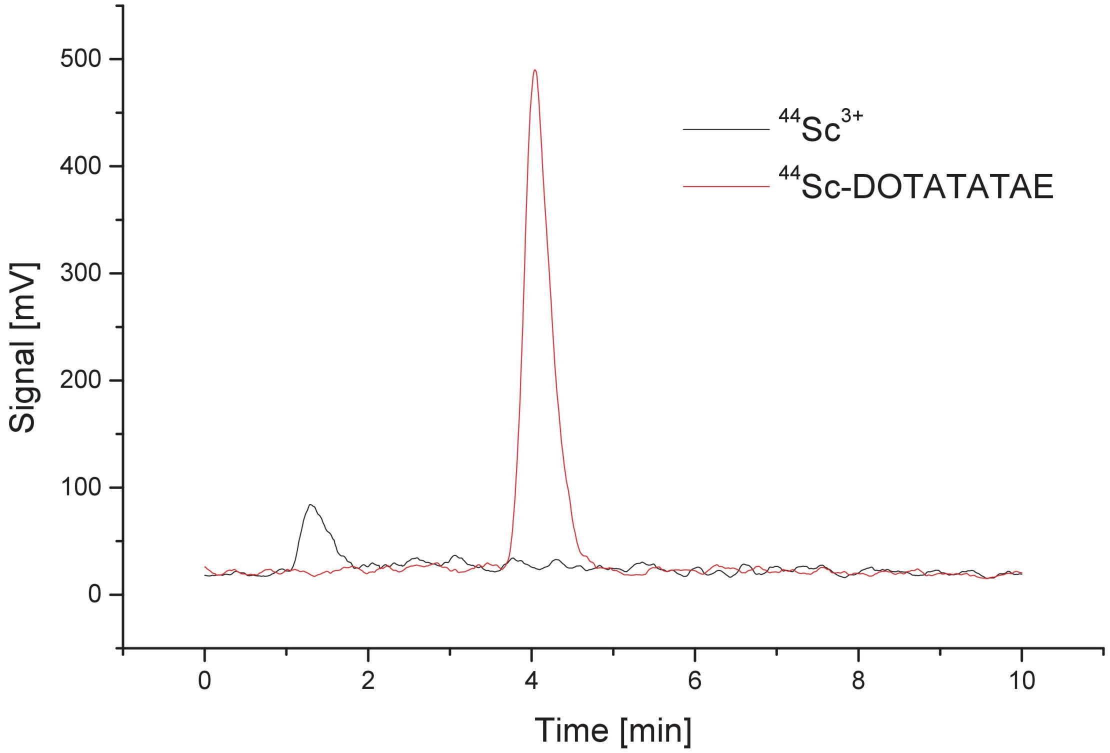

Figure 4S. Resolution test for ^{44}Sc (t_r=1.7 min) and ^{44}Sc-DOTATATE (t_r=4.2 min). Conditions: HPLC system Shimadzu AD20 with UV-Vis and radiometric detector GabiStar , Phenomenex Gemini C18 column (150 mm × 4.0 mm i.d., 5 μm), with 375:115:0.5 (v/v/v) water:acetonitrile:trifluoroacetic acid as a mobile phase and 1.5 mL/min flow rate.

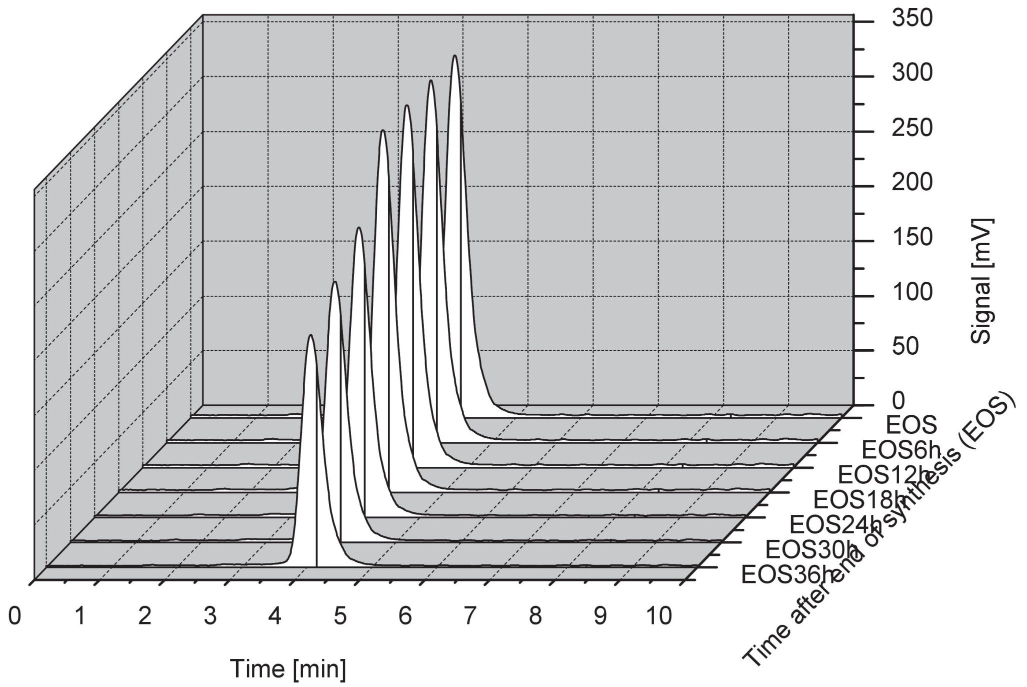

Figure 5S. Stability test for ^{44}Sc-DOTATATE within 36 h after end of synthesis (EOS). Injection volume (2-100 μL) was adjusted to the decay of radioisotope to ensure the comparability of signals.

Table 1S. ^{44}Sc labeling yields for DOTATATE at different ligand concentrations. t=95°C, acetate buffer pH=4.0, 30 min.

Ligand amount (nmol)	Yield [%]
28.6	99.6
14.3	99.6
7.1	99.5
3.6	99.5
0.71	99.5
0.36	99.3
0.07	95.5

Figure 6S. γ-spectra of produced ^{44}Sc. Radionuclidic purity >97%.

Aquisition: detector CANBERRA HPGe model: BE2825; thickness: 25.5 mm; active area: 2800 mm^2; window: Carbon Epoxy 0.6; measurement life time: 3568 s; measurement dead-time: 0.8%; frontal measurement; detector-sample distance: 5.5 cm

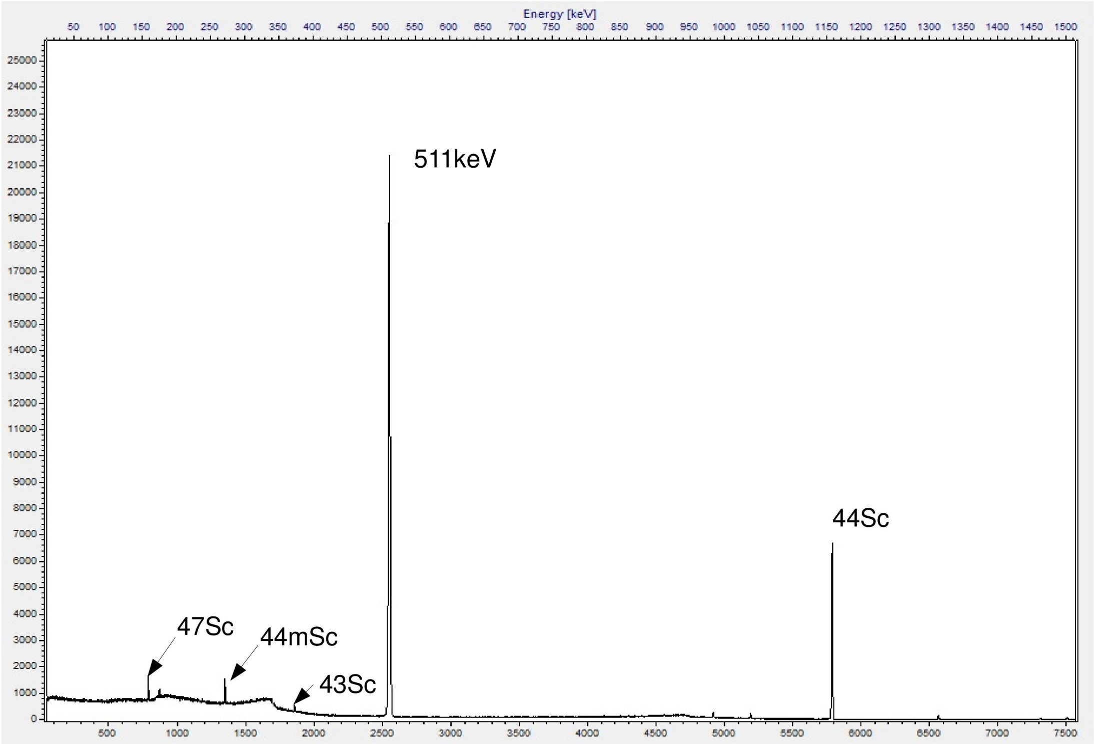

Experimental data

					Labeling				
Irradiation date	Target	mass [mg]	beam current	Irradiation time	Estimated Activity EOB [Mbq]	Activity processed [Mbq]	Separation mode	Separation yield[%]	Labeling RCY[%]
30.11.2017	CaCO3	89,005	~10 uA	~2h	179,1	127	Filtration	82,3	98,1
6.12.2017		87,232			183,6	125	Filtration	78,5	99,0
20.12.2017		83,341			207	138	Nobias	91,7	99,5
21.02.2018		90,831			163,8	91,3	Nobias	97,4	99,1
21.03.2018		90,157			205	141	Filtration	87,4	99,0
28.03.2018		89,901			170	95	Filtration	47,8	98,5
18.04.2018		89,708			200	142,7	Filtration	96,1	98,7
25.04.2018		87,465			152,3	87	Nobias	94,4	98,9
7.05.2018		90,144			188	114,4	Filtration	67,7	98,2
16.05.2018		86,689			177	116	Nobias	92,4	99,6
21.05.2018		88,799			151,8	88,6	Nobias	99,0	99,5

Chemical purity [mg/L]

Sample	Al	Ca	Cr	Cu	Fe	Mn	Ni	Pb	Zn
Nobias 1	0,013	0,302	0,001	0,002	0,003	0,014	0,025	0,063	0,037
Nobias 2	0,004	0,617	0,002	0,021	0,007	0,014	0,007	0,020	0,018
Nobias 3	0,010	0,105	0,001	0,049	0,006	0,014	0,006	0,007	0,039
Average	0,009	0,341	0,001	0,024	0,005	0,014	0,013	0,030	0,031
